# 

**Published:** 1876-04

**Authors:** 


### TABLE OF CONTENTS.

ORIGINAL COMMUNICATIONS—

Loss of Crystalline Lens by Blow on Eyeball. By Thomas H. Kenan, 2d Assistant Physician Georgia Lunatic Asylum......... ... 193
Partial Occlusion of the Vagina and an Almost Imperforate Hymen, in Case of a Lunatic, with Recovery after Operation. By Thos. H. Kenan, 2d Assistant Physician Georgia Lunatic Asylum...... 194
Fractured Skull—Trephine in Case of an Insane Epileptic. By Thos. H. Kenan, 2d Assistant Physician Georgia Lunatic Asylum...... 195
Cases in Practice. By A. R. Kilpatrick, M.D., Navasota, Texas..... 196
The Opium Habit. By A. A. Lyon, M.D., Shreveport, Louisiana.... 200
Observations in Practice. By Dr. W. L. Lipscomb, Columbus, Miss. 204

SELECTIONS—

Diphtheria and Filth........................................ 205
Dietetics.................................................... 209
Salicylic Acid in Aural Discharges. By Julian J. Chisolm, M.D..... 212
On Piles, their Diagnosis and Treatment. By Alfred Cooper, F.R.C.S. 215
Extract from Paper by Wm. Porter, M.D., on Nasal Catarrh........ 219

EXTRTCTS AND GLEANINGS.

Atropia in Phthisical Sweating—223. Diagnosis of Iritis—225. Indications for Counter-Irritation—226. The Lungs—Breathing; Treatment of Uterine Tetanus—227. Pruritus Pudendi in Pregnant Women—228. Treatment of Constipation and Diarrhœa with Water and Tincture of Camphor—229. Treatment of Intestinal Obstruction; Bromide of Potassa in Infantile Convulsions—230. Case of Rheumatism with Treatment—231. Extensive Burns—232. Medicated Ice in Scarlatina—233. On the Employment of Iodoform in Vaginismus; Uterine Hemorrhage—234. Diphtheria—235. Treatment of Lead Colic in the Paris Hospitals—236. Ammonia in Rheumatism; Chloral—237. Treatment of Gonorrhœa—238. A Question on the Physiology of the Brain; Treatment of Albuminuria During Pregnancy—239. On the Treatment of Phagedenic Gangrenous Venereal Sores; Bromide of Lithium—240. Baths of Chloral Hydrate in Confluent Small Pox; Hypodermic Injection of the Neutral Sulphate of Atropine in a Case of Reflex Contraction—241. Treatment of Eczema in Children; Delirium Tremens—Treatment with Lupuline; Counteracting the Nausea of Opiates with Belladonna—242. Scientific Gleanings—243, 244.

EDITORIAL AND MISCELLANEOUS.

Imposed Upon; W. A. Townsend; Thomas Pullum & Co.......... 245
Truths and Facts............................................ 246
Advertising—Our Colaborer Speaks Again.................... 251
Bibliographical.............................................. 254

